# Management of postmenopausal osteoporosis

**DOI:** 10.1210/endrev/bnag006

**Published:** 2026-02-26

**Authors:** Ian R Reid, Mark J Bolland

**Affiliations:** Department of Medicine, University of Auckland, Auckland 1142, New Zealand; Department of Medicine, University of Auckland, Auckland 1142, New Zealand

**Keywords:** osteoporosis, osteopenia, fracture, bisphosphonates, drug treatment

## Abstract

Postmenopausal women experience ongoing loss of bone mass, with resulting increases in the risk of fracture. This review describes the nature of postmenopausal bone loss, the definition of osteoporosis, and the current status of fracture risk estimation, which is pivotal in osteoporosis management. Important lifestyle measures include taking a balanced diet to maintain a healthy weight throughout life, safe physical activity, not smoking, and moderating alcohol intake. Severe vitamin D deficiency accelerates bone loss so should be avoided. Falls prevention becomes increasingly important with age, since falls cause most fractures. Pharmaceuticals to increase bone mass and prevent fractures either act by inhibiting bone resorption or by stimulating bone formation. Bisphosphonates are the most widely used antiresorptives, often taken as weekly oral doses. The intravenous bisphosphonate, zoledronate, has a long duration of action with effects on bone turnover, density, and fractures over a decade after a single dose. It is increasingly used in both prevention and treatment of osteoporosis. Denosumab is effective in preventing fractures but has a rapid offset of effect after its cessation. Some anabolic agents act via the PTH1 receptor, producing substantial increases in spine bone density but are not yet proven to prevent hip fractures. Romosozumab is a monoclonal antibody directed at sclerostin. It has both anabolic and antiresorptive effects and shows broad antifracture efficacy. Anabolics are used for 1 to 2 years in those with high fracture risk, before transition to long-term antiresorptive therapy. Treatment sequence options are discussed but more research is needed to establish which provide optimal fracture reduction.

## Essential points

- Osteoporotic fractures are an increasing public health burden as a consequence of increasing longevity
- Lifestyle factors are important throughout life, including maintaining a healthy weight, being physically active, avoiding smoking, and moderating alcohol intake
- Prevention of severe vitamin D deficiency is important particularly among the frail elderly
- Estimation of fracture risk is pivotal in assessing a person at risk of fractures
- Bisphosphonates are common first-line agents, zoledronate being attractive because of its safety and infrequency of administration
- Those at high risk of fracture often receive a course of an anabolic drug initially, before transition to ongoing antiresorptive treatment
- Osteoporosis requires lifelong management, usually involving a sequence of agents, and sometimes including drug holidays

## The osteoporotic process

Osteoporosis is a condition “characterized by low bone mass and microarchitectural deterioration of bone tissue, with a consequent increase in bone fragility and susceptibility to fracture” (1). In both sexes, bone loss with advancing age is universal. In women, this is more dramatic and is closely related to the abrupt fall in circulating estrogen levels after the menopause, which causes increased bone turnover with an excess of bone resorption over formation. The bone mass of older women is progressively impacted by this bone loss, but also dependent on their peak bone mass, body weight, intercurrent illnesses, medications (particularly glucocorticoids), smoking, alcohol intake, and exercise. With advancing age, falls risk also increases and is a further important contributor to fractures. The consequence of these changes is a progressive increase in total fracture risk from 8/1000 patient-years aged 50 to 54, to 16/1000 patient-years aged 70 to 74, and to 31/1000 patient-years aged 80 to 84 years, in a multiethnic cohort of United States women (2). Age and bone mineral density (BMD) are important, independent determinants of fracture risk. Thus, bone loss is appropriately regarded as an integral part of aging, affecting all older adults who, therefore, need to be aware of their fracture risk as they age, and to consider if and/or when interventions to reduce that risk are appropriate.

## Defining osteoporosis

The qualitative definition of osteoporosis given in the previous paragraph was produced by a consensus development conference in 1993 and remains widely used and accepted. It captures the key features of osteoporosis for both patients and clinicians. Subsequently, quantitative definitions were proposed, based on fracture history, BMD, or estimates of fracture risk. Although these quantitative measures are important in defining thresholds for treatment, which should vary with the cost, efficacy, and safety of the available medicines, such variability is less acceptable in defining a diagnostic category. This difficulty is now very clear with the BMD definition of osteoporosis (a T-score of < −2.5), put forward by a World Health Organization (WHO) working group in 1994 (3). Since that time, when the science of osteoporosis was embryonic, it has become apparent that 80% of postmenopausal women who fracture do not meet this definition (4-7), even though they demonstrably suffer from bone fragility and meet the qualitative definition of osteoporosis. This conflict between the BMD definition of osteoporosis and its clinical presentation probably plays a large part in the low uptake of osteoporosis medications because patients and doctors are reluctant to take or provide treatment for a condition not considered to be present (8). Furthermore, BMD measurements are not freely available to much of the world's population, depriving them of the possibility of osteoporosis being diagnosed and appropriate treatment provided. Accordingly, the WHO is now reappraising its endorsement of a BMD-based definition of osteoporosis (https://extranet.who.int/dataformv6/index.php/832951?lang=en).

To address this problem, we suggest that clinicians diagnose osteoporosis based on the qualitative definition put forward in 1993. Increased “susceptibility to fracture” can be determined from a patient's calculated fracture risk. The level of risk that is considered “osteoporotic” might vary over time and with location but should broadly align with local prescribing practice. This will, in turn, be impacted by medication costs and availability. Although epidemiologists might find this unsatisfactory, patients at increased fracture risk will be diagnosed with osteoporosis and then offered appropriate treatment for it, which fits with practice across most other areas of medicine. This is likely to achieve greater rates of treatment uptake than the current situation, in which a patient is told they do not have osteoporosis (by BMD criteria) but that they should take medications anyway.

## Overview of osteoporosis management

The broad approach to a patient at risk of fractures is presented in Table 1. Strategies can be directed at the prevention of bone loss in younger individuals at low risk of fractures, or at reducing fracture risk in those in whom it is already elevated. Both lifestyle interventions and medications can be employed in each of these contexts. Falls prevention is an important aspect of fracture prevention at any age. The US Preventive Services Task Force has recently reaffirmed its recommendations that postmenopausal women aged older than 65 years, and younger women with clinical risk factors should be screened for osteoporosis. They made no recommendations for men (9, 10). Screening would usually be done by calculating fracture risk, supplementing this with BMD measurement if appropriate (Table 1). Individuals whose BMD is lower than would be expected for their age, weight, and other clinical risk factors should have investigations looking for conditions that might cause bone loss (eg, inflammatory disorders, malignancy, hypogonadism, endocrinopathies, osteogenesis imperfecta, medications) (11).

**Table 1:** Framework for osteoporosis management

**Prevention of bone loss**
**Lifestyle** weight-bearing exercise maintain normal BMI not smoking alcohol ≤2 drinks daily regular, safe sunshine exposure **Medication** vitamin D supplements in those at risk of severe vitamin D deficiency with low sunlight exposure (eg, high latitude, frail elderly, veiled) or dark skin very infrequent zoledronate in motivated individuals hormone treatment in women with menopausal symptoms
**Assessment of fracture risk**
Assess with validated risk calculator, such as FRAX or Garvan, with or without BMD. Assess in women in 50’s with risk factors or in most women in 60’s. If MOF 10-year risk >10%, consider adding BMD measurement if it would lead to a change in management.
**Drugs to reduce fracture risk**
**MOF 10-year risk >10%-15%** Oral bisphosphonate up to 5 years, then drug holiday (risedronate 1 year, alendronate 1-2 years) Zoledronate 5 mg 18-monthly × 4 doses, then less frequently (eg, 3 yearly). Less frequent dosing from the outset might be acceptable in those at lower fracture risk. Denosumab suitable for those who cannot take bisphosphonates, but it requires high adherence and an exit strategy Estrogen or SERMs are further options, but SERMs are less effective **MOF 10-year risk >20%-30%** Initiate treatment with anabolic agents (eg, teriparatide, abaloparatide, romosozumab), if available and cost-effective Change to antiresorptive treatment following course of anabolic
**Monitoring treatment response** Regularly confirm correct dosing procedure for oral bisphosphonates Consider confirming suppression of bone turnover markers 3-6 months following initiation of oral bisphosphonates Confirm regular dispensing of medication, particularly denosumab Consider repeat BMD 3-5 years after treatment initiation, especially if it will lead to a change in management
**Possible treatment sequences** Continue cycles of oral bisphosphonates and drug holidays as long as fracture risk justifies treatment Zoledronate 18-monthly, transitioning to maintenance doses every 3 years as long as fracture risk justifies treatment Zoledronate for prevention of bone loss dosing at 5- to 10-year intervals Denosumab must be dosed every 6 months. If stopping denosumab, usually transition to a bisphosphonate. After an anabolic, transition to an antiresorptive. Second courses of teriparatide and romosozumab have been described but antifracture efficacy unknown

Abbreviations: BMD, bone mineral density; BMI, body mass index; MOF, major osteoporotic fracture; SERM, selective estrogen receptor agonist.

## Estimation of fracture risk

Although BMD is highly predictive of fracture (12), it is but 1 of many risk factors—age, body weight, falls risk, and fracture history that are of comparable importance in determining fracture risk. These risk factors can be used in the calculation of fracture risk, providing a single parameter that can guide treatment decisions. The development of fracture risk calculators has been a critical step forward in osteoporosis management over the last 20 years. A number of calculators are available, the FRAX Fracture Risk Assessment Tool being the most widely used, though the algorithm underpinning it has not been published. Clinical variables (age, sex, weight, height, personal and parental history of fracture, smoking, alcohol, glucocorticoid use etc.) are entered, and the 10-year risk of hip fractures and major osteoporotic fracture (MOF) are calculated, adjusted for the competing risk of death. Multipliers are now available that allow for other risk factors to be added to the model (13, 14). In older postmenopausal women not receiving bone-active drugs, the FRAX risk of hip fracture doubles every 5 to 6 years (15). The core model is derived from a number of prospective observational studies but fracture risk estimates are calibrated for the rates of hip fracture and death of the local populations.

The Garvan Fracture Risk Calculator was developed in Australia. It does not adjust for ethnicity, which effectively limits its use to White populations. It uses a smaller group of clinical risk factors, including the number of previous fractures and the number of falls in the last year, which are important determinants of fracture risk. Both of these calculators can be used with or without BMD measurements, with FRAX performing comparably with or without BMD in a Canadian cohort (16). The Q-Fracture calculator accesses health databases for other data that inform fracture risk (17).

Two important differences between the FRAX and Garvan calculators are the timeframe of risk prediction and incorporation of competing risk of mortality. FRAX gives estimates of 10-year fracture risk after accounting for the competing risk of mortality, whereas Garvan gives 5- and 10 year-risk estimates without this adjustment.

Three- to 5-year estimates are clinically relevant in older patients because substantial treatment benefits with antiosteoporosis medicines (AOMs) occur within this timeframe, which also aligns with current recommendations for initial duration of AOMs. Incorporating competing risk of mortality into risk estimates leads to lower predicted 10-year risks from FRAX. These lower risk estimates and long prediction timeframe obscure the substantial short-term benefits from treatment. This may, in turn, lead to undertreatment of older individuals at high risk of fracture (18).

Because there are substantial differences in mortality and hip fracture rates between various national and/or ethnic populations, accounting for these variables in FRAX substantially impacts on fracture estimates. Recently, there has been controversy regarding the use race or ethnic labels in medical practice, and the American Society of Bone and Mineral Research convened a group which concluded that using a single population-based calculator for the United States “will reduce differences in treatment qualification and may ultimately enhance equity and access to osteoporosis treatment” (19). However, ignoring the substantial differences in fracture risk associated with race and ethnicity is likely to result in inaccurate estimates of fracture risk, with flow-on clinical effects of treatment of individuals at low risk for little benefit and nontreatment of individuals at high risk, leading to unnecessary fractures. A working group from the International Osteoporosis Foundation reached a similar conclusion and recommended the retention of ethnic and race-specific FRAX models (20).

## Overview of interventions to prevent fractures

Fracture prevention is the primary goal in osteoporosis management. Strategies to achieve this could be implemented in early life through maximization of bone density, in midlife through minimization of bone loss (particularly after the menopause), or by increasing/stabilizing bone mass in older adults who already have osteoporosis and a high fracture risk. Nutrition, lifestyle and pharmaceutical measures have all been investigated to this end, with variable success. Enormous effort has been invested in assessing the role of calcium intake on bone, with little evidence that increasing calcium intakes makes a clinically significant difference to fracture risk at any age (reviewed subsequently). Body weight, however, is closely related to both BMD and fracture risk, so nutritional policy should be targeted toward taking a balanced diet that avoids underweight throughout life. Hip fracture risk, in particular, rises steeply when body mass index is <20 (21, 22). Protein intake may be of key importance to fracture risk in older individuals (23), as found in a recent trial of dairy product supplementation in older adults in residential care (24). Smoking and high alcohol intakes are risk factors for fracture and should be avoided. In older adults, most fractures result from falls, and a history of falls increases future fracture risk by 40% to 50% (25). Falls can be prevented by multifaceted interventions including changes to the home environment (eg, handrails, removal of trip hazards), vision optimization, minimization of sedative use, and monitoring antihypertensive drugs to prevent postural hypotension (26, 27). Exercise programs incorporating impact, high-intensity resistance, and balance training can reduce falls, maintain or improve bone mass, and prevent fractures (28, 29).

Circulating 25-hydroxyvitamin D concentrations reflect both nutrition and lifestyle, and their role in osteoporosis management will now be considered. This is followed by review of available pharmaceutical interventions, focusing on areas of new data or controversy. A summary of registered indications and doses of AOMs has been published recently (30). A summary of the clinical approach to the patient at risk of osteoporosis is provided in Table 1.

## Vitamin D

Vitamin D is misnamed—it is not an essential dietary constituent as that name implies but instead is the biologically inactive precursor of an endocrine system that primarily regulates the intestinal absorption of calcium and phosphate. It is formed in the skin as a result of the action of ultraviolet light, and, along with its metabolites, circulates bound to vitamin D-binding protein. Its principal circulating form is 25-hydroxyvitamin D, and this metabolite is used clinically to assess adequacy of vitamin D status. Severe, prolonged deficiency of vitamin D results in hypocalcemia ± hypophosphatemia and secondary hyperparathyroidism, leading to undermineralization of bone. This results in the clinical syndromes of rickets in children and osteomalacia in adults. Accordingly, vitamin D supplements are critical in the treatment and prevention of these conditions.

After the use of vitamin D in treating osteomalacia was established in the 1920s, it came into use in adults with osteoporosis, before there was any trial evidence to support this practice. However, it has now been extensively examined over the past 20 years, in a series of large trials reporting on bone and other endpoints (31-34). The great majority of these studies has been neutral, showing no effects on BMD or fractures (35-37), although the D-Health trial suggested a beneficial fracture effect in the last of its 5 years (38). One large trial of vitamin D plus calcium in severely vitamin D-deficient women in nursing homes did find a reduction in fractures (39, 40), but this was not reproduced with a lower dose of vitamin D in a similar population (41), nor with an intervention of low-dose vitamin D (400 IU/day) plus calcium in a very large trial in community-dwelling women (42). These different outcomes suggest that there is a 25-hydroxyvitamin D threshold above which vitamin D supplements are without benefit. In the light of this evidence, the Endocrine Society has withdrawn its previous high thresholds for defining both vitamin D “sufficiency” (ie, > 75 nmol/L) and “insufficiency” (ie, 50-75 nmol/L) (43).

The absence of a vitamin D effect on BMD in the community contrasts with a dramatic positive effect on BMD in those with osteomalacia (44), suggesting that this is a threshold effect. A prospective study to identify this threshold found that increases in BMD after vitamin D supplements were only seen in those with baseline 25-hydroxyvitamin D < 30 nmol/L (45). A post hoc analysis of an earlier Scottish trial of vitamin D supplementation confirmed this finding (46). Therefore, vitamin D supplements may be a useful therapeutic intervention in those with 25-hydroxyvitamin D < 25-30 nmol/L, and maintaining 25-hydroxyvitamin levels comfortably above this level is a sensible public health goal.

Determining the role of vitamin D in osteoporosis management is complicated by its administration to both intervention and placebo groups in most clinical trials. This has sometimes led to the assumption that it is required for osteoporosis medications to be effective. This assumption should be critically reappraised because positive effects of estrogen and bisphosphonates on BMD and fractures have been found in trials not coadministering vitamin D (47-49). Potent antiresorptive drugs given to individuals with severe vitamin D deficiency do sometimes result in symptomatic hypocalcemia, so vitamin D status needs to be considered before using such agents. The need for vitamin D supplements in osteoporosis management is dependent on the expected 25-hydroxyvitamin D level in a particular patient, which will depend on season, lifestyle, body weight, skin color, and environment. In some countries, measurement of 25-hydroxyvitamin D is a routine part of the assessment of a patient with osteoporosis, whereas in others this is regarded as an unnecessary expense since the cost of supplements is often much less than the cost of the blood test, and the need for vitamin D supplementation can usually be determined clinically. Use of supplements should be individualized accordingly and seldom need to exceed 400 to 1000 IU/day, which will raise 25-hydroxyvitamin D substantially above the 25 nmol/L threshold (46). Higher doses may be needed in patients with malabsorption or obesity. At these doses, vitamin D supplements simply replace cutaneous synthesis as a source of substrate for the vitamin D endocrine system. The use of much higher doses (eg, > 2000 international units daily) overrides the normal homeostatic checks, raises 25-hydroxyvitamin D to much higher levels and, in some studies, has been associated with loss of BMD and increases falls and fractures (50-52). These adverse effects on bone are likely related to vitamin D-mediated stimulation of bone resorption (53).

## Antiresorptive agents

**Figure 1:**
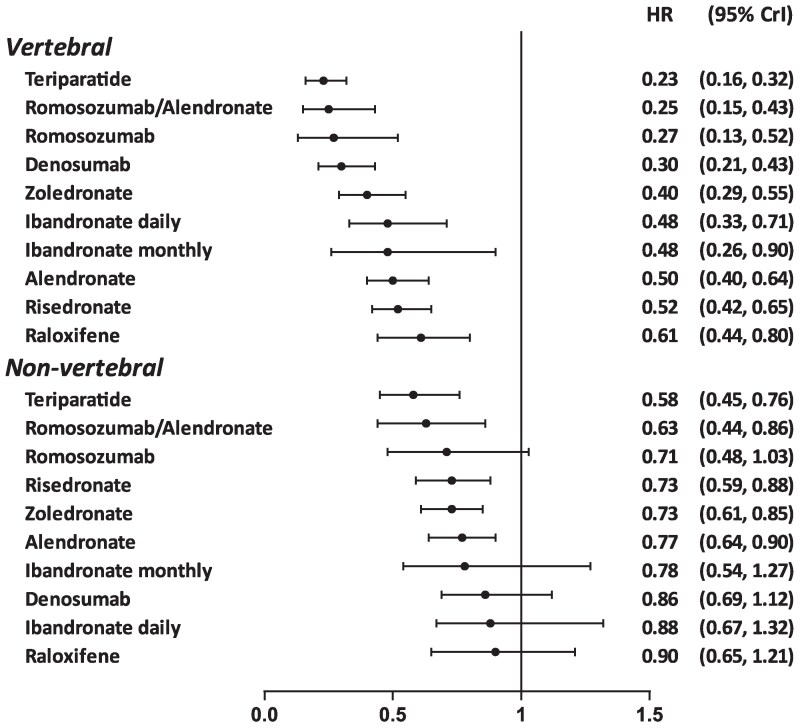
Forest plot for vertebral and nonvertebral fractures based on a network meta-analysis commissioned by the National Institute of Health Research (54). Hazard ratios (HR) and 95% credible intervals for the effects of each treatment relative to placebo are presented. Abaloparatide was not included in this analysis. Figure reprinted from: Reid IR & Billington EO, Lancet 399:1080-1092, 2022 (30), with permission.

Bone is a connective tissue consisting of bundles of type I collagen fibers laid down by osteoblasts and resorbed by osteoclasts. Postmenopausal bone loss primarily results from an increase in osteoclast activity as a consequence of the dramatic fall in circulating estrogen levels around menopause. This suggests that drugs that inhibit osteoclasts (ie, antiresorptive agents) are highly appropriate for managing postmenopausal bone loss. Bisphosphonates and denosumab are the 2 main classes of antiresorptive drugs, though estrogen and selective estrogen receptor modulators are also primarily antiresorptive in their actions. The fracture effects of these agents are mostly well established (Fig. 1), but new data continue to emerge regarding their safety.

### Oral bisphosphonates

The bisphosphonate nucleus consists of 2 phosphate groups joined through a linking carbon atom, to which 2 further side groups are also attached. These side groups distinguish the members of the class and determine their critical properties—avidity of binding to the bone surface (55) and potency of inhibition of their target enzyme, farnesyl pyrophosphate synthase (56).

Bisphosphonates have a unique pharmacology. They are negatively charged ions at physiological pH and have an oral bioavailability of only about 1%. Taking bisphosphonates fasting with water alone is critical to achieve even this absorption, and co-ingestion with food or positively charged ions (eg, calcium, antacids) effectively reduces bioavailability to zero. Absorbed bisphosphonate is rapidly taken up onto the bone surface or excreted into the urine, so circulating levels are close to zero 24 hours after intravenous administration. The half-life of bisphosphonate in bone is measured in years, though some drug is buried under newly formed bone so is not necessarily available to inhibit currently active osteoclasts. Surface bisphosphonate is mobilized during bone resorption and taken into osteoclasts by pinocytosis. In the osteoclast, bisphosphonates inhibit a key enzyme (farnesyl pyrophosphate synthase) in the mevalonate pathway, interfering with the cell's cytoskeleton, leading to reduced bone resorption and osteoclast apoptosis.

#### Efficacy

The efficacy of oral bisphosphonates has been extensively reviewed previously and will only be briefly discussed here. Alendronate increases total hip BMD to a plateau about 3% above baseline, after 3 to 4 years treatment (57). BMD increases tend to be larger at sites rich in trabecular bone, such as the spine, because bone turnover is higher in trabecular bone. Risedronate produces smaller increases in BMD (58) but the antifracture effects of these agents are comparable (Fig. 1). Oral ibandronate has similar effects on vertebral fracture to alendronate and risedronate but has not been shown to significantly reduce nonvertebral fracture risk (Fig. 1). Alendronate and risedronate both reduce hip fracture risk by about 40% (54).

The avid binding of bisphosphonates to the bone surface makes these agents among the longest acting of all pharmaceuticals. However, binding avidity varies among the individual bisphosphonates (55). As a result, risedronate effects on markers and BMD show offset 6 to 12 months after drug cessation (59, 60), but alendronate effects on markers, BMD, and fractures persist rather longer. Thus, after alendronate treatment, hip BMD returns to near baseline after about the same period off drug as that on treatment (57, 61), and hip fracture rates during drug holidays exceeding 12 months are higher in those previously on risedronate compared with previous alendronate users (62). The effects of monthly doses of ibandronate on markers and BMD are intermediate between those of alendronate and risedronate (60).

#### Safety

The most common side effect of oral bisphosphonates is gastrointestinal symptoms, resulting in drug discontinuation in 14% of patients in 1 study (63). These drugs are contraindicated in those with active disease in the esophagus, stomach, and upper small bowel. Patients are advised to remain upright for at least 30 minutes after dosing to minimize the risk of gastroesophageal reflux. Intravenous bisphosphonates are more appropriate in patients with gastrointestinal problems. In patients with severe vitamin D deficiency (ie, 25-hydroxyvitamin D < 25 nmol/L), potent antiresorptive agents can cause hypocalcemia, although this is more common with parenteral agents such as zoledronate or denosumab. Oral bisphosphonates are not recommended in patients whose creatinine clearance is <35 mL/min, and such patients were excluded from the pivotal trials of these drugs. Only observational data address this safety question, and these are contradictory (64, 65). In 1 study of matched patients with chronic kidney disease (CKD) stage 3b-5, those using bisphosphonates had a 15% increase in their risk of CKD progression (66). Decisions regarding osteoporosis management in CKD are complex, and joint management with a nephrologist is often needed. A review of the limited options for managing osteoporosis in CKD has recently been published (67). Osteonecrosis of the jaw (ONJ) and atypical femoral fractures (AFF) are much rarer adverse events associated with antiresorptives and will be discussed next.

### Zoledronate

Zoledronate is the most potent and long-acting bisphosphonate in clinical use. It is only available as an intravenous infusion, although oral formulations have been studied and are poorly tolerated and less sustained in their antiresorptive effects (68). In early studies, zoledronate was given as a slow intravenous injection, but this caused renal injury in some patients with preexisting renal impairment, so it was developed as a 15-minute infusion at a volume of 100 mL (69). The optimal dosing regimen for zoledronate in osteoporosis is unknown. The phase 2 trial compared doses between 0.25 and 4 mg at intervals of 3 to 12 months (70). These regimens were indistinguishable with respect to their effects on both markers and BMD. It was decided to use annual dosing in the phase 3 trial, and a new dose of 5 mg was chosen to distinguish it commercially from the oncology preparation of 4 mg. Unlike other antiresorptive drugs, substantial new trials of zoledronate have been published in recent years that require a more detailed description here.

#### Efficacy in osteoporosis

The pivotal phase 3 trial randomized 7736 osteoporotic women to receive infusions of zoledronate (5 mg) or placebo annually for 3 years. Zoledronate reduced vertebral fracture risk by 70%, nonvertebral fractures by 25% and hip fractures by 41% (*P* < .001 for all) (71). At 3 years, the placebo group was discontinued, and some patients from the zoledronate group were rerandomized to continue zoledronate or to crossover to placebo for a further 3 years. Vertebral fracture risk was lower in those continuing zoledronate compared with those crossing over to placebo, but there was no between-groups difference in nonvertebral fractures, though fewer than 1000 participants completed follow-up to 6 years (72). However, after 6 years of annual dosing, drug discontinuation was followed by <1% decline in BMD over the following 3 years, and fracture rates were similar in those who had either 6 or 9 years of zoledronate (73).

A second phase 3 study was carried out in 2127 patients (76% women) with a hip fracture in the preceding 90 days (74). Annual dosing with zoledronate reduced the risk of new clinical fracture by 35%, and death (a prespecified safety endpoint) was reduced by 28% (*P* = .01). A post hoc analysis in participants from these two phase 3 studies who received only 1 infusion (of zoledronate or placebo) found a 32% reduction in clinical fracture and a 68% reduction in vertebral fracture in the zoledronate-treated patients over 3 years, suggesting that a single dose over 3 years might have comparable efficacy to annual dosing (75).

#### Efficacy in osteopenia

Because the phase 2 study had not identified an optimal dose and frequency for zoledronate administration, Grey et al compared the long-term effects of single doses of 1 mg, 2.5 mg, and 5 mg on bone turnover markers and BMD in osteopenic postmenopausal women (76). They found that the effects of the 3 doses at 6 to 12 months were comparable, but that longevity of the effects was markedly dose-dependent. Beyond 18 months, the BMD effects of a single 5-mg dose were less than previously reported with annual dosing. Accordingly, in a subsequent 6-year trial of zoledronate 5 mg in osteopenic women aged >65 years, we chose 18 months as the dosing interval (77). The antifracture efficacy of this regimen was of a similar magnitude to that demonstrated in the phase 3 trials in osteoporosis (71, 74), and this was independent of baseline participant characteristics, including age, BMD, and fracture risk (78), suggesting this regimen is effective in a wide range of postmenopausal women. There was a progressive decline in the fracture rate in the zoledronate group over the 6-year trial, whereas the placebo group showed a progressive rise in fracture rate. The rate ratio (ie, the ratio of fracture rates in the 2 groups) for nonvertebral fragility fractures in years 4 through 6 was 0.40 (95% CI, 0.27-0.58), indicating that when used long-term, zoledronate is among the most effective agents for prevention of nonvertebral fractures (79).

We have now studied the zoledronate group from this 6-year study (77) to 10 years without further treatment (80). After the last zoledronate infusion at 4.5 years, fracture rates remained stable until about year 8, but then increased significantly. However, the cumulative incidence of fracture remained below the extrapolation of that from the placebo group, indicating that some antifracture efficacy persists even without further zoledronate dosing (80). These data suggest that low fracture rates can probably be maintained with infrequent dosing (eg, 3-4 yearly [ie, at the time fracture risk increased here]) after earlier higher frequency dosing.

#### Prevention of postmenopausal bone loss

**Figure 2:**
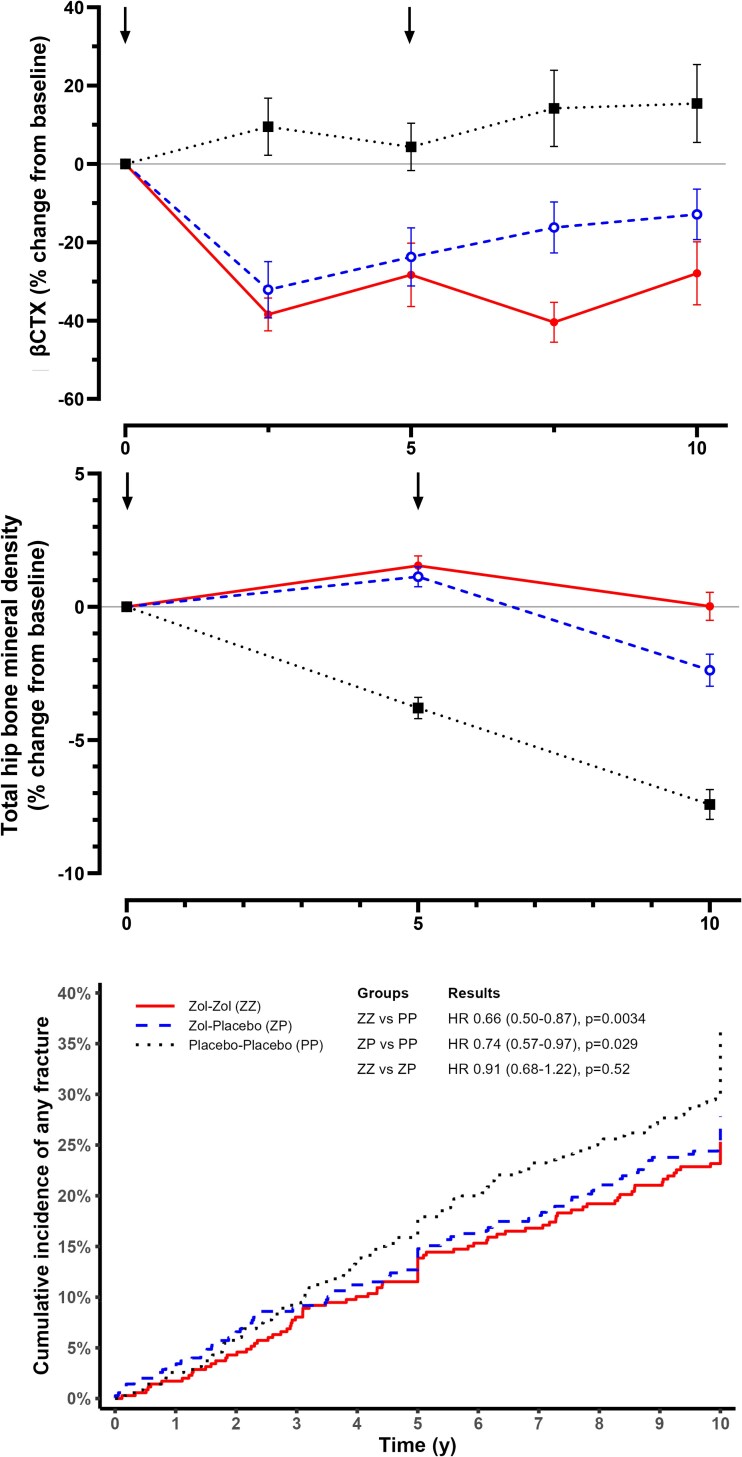
Effects of placebo or zoledronate every 5 years, or zoledronate once at baseline on bone resorption, BMD, and total fractures, in a trial of 1054 early postmenopausal women aged 50 to 60 years, studied over 10 years. Percent change in β-CTX and BMD are presented as mean and 95% CI. The cumulative incidence of any fragility fracture over time by treatment group are presented as hazard ratios. Arrows indicate infusion times at baseline and 5 years). Abbreviations: βCTX, β-C-terminal telopeptide of type I collagen; placebo-placebo, placebo at baseline and at 5 years; Zol-placebo, zoledronate at baseline and placebo at 5 years; Zol-Zol, zoledronate at baseline and 5 years. Based on data from Bolland et al (48).

The initial trial development program for zoledronate included a study demonstrating significant beneficial effects on BMD and markers from a single dose over 2 years (81), resulting in the registration of “prevention of postmenopausal osteoporosis” as an indication in the United States. Subsequently, it was shown that a single dose of zoledronate 5 mg could maintain BMD near baseline for almost 10 years (82). To determine whether these effects prevent fractures, Bolland et al assessed the effects on fractures of zoledronate in postmenopausal women aged 50 to 60 years with baseline BMD T-scores between zero and −2.5 (48). The study compared fracture incidences over 10 years among placebo, a single 5-mg zoledronate dose, and 5-yearly zoledronate dosing. Both 5-yearly and 10-yearly zoledronate prevented fractures—relative risks of vertebral fracture 0.56 and 0.59, and of any fracture 0.70 and 0.77, respectively. Antifracture efficacy was not significantly different between the zoledronate dosing regimens, but BMD and marker effects beyond year 5 were greater in the 5-yearly dosing group (Fig. 2). This study establishes an effective and inexpensive regimen for prevention of fractures in postmenopausal women, which is likely to be more acceptable to many early postmenopausal women than weekly tablets over many years. The increasing antifracture effect over the 10-year study period suggests that, with further 5- to 10-yearly dosing to maintain BMD, effects on fractures will be maintained or even increased over time (83).

#### Which zoledronate regimen to use?

There is now robust evidence for fracture prevention with zoledronate infusions at intervals between 1 and 10 years. The reductions in fracture risk with 5- to 10-yearly dosing are remarkably similar to those found in the studies in older women where zoledronate was administered every 12 to 18 months (71, 74, 77). Does this mean that these regimens can be used interchangeably in patients at both low and high risk of fracture? Probably not. It would be unwise to assume that very infrequent dosing is optimal for fracture prevention in older women because we have found that fracture rates start to rise in women aged >65 years when zoledronate is withheld for more than 4 years after regular dosing (80). However, zoledronate dosing every 18 months in older women with osteoporosis is likely to be successful because there was a substantial overlap in clinical features (age, fracture risk, prevalent fractures) between the women in that trial and those in the phase 3 osteoporosis studies. Importantly, fracture prevention with zoledronate was independent of these and all other baseline characteristics (78). The trial of 18-month dosing demonstrated fracture prevention over 6 years, which was sustained for several more years posttrial, achieved numerically greater risk reduction for nonvertebral fractures than the phase 3 studies, particularly in years 4 through 6. Zoledronate administration every 18 months is cheaper and more convenient than annual dosing. Therefore, a regimen of 4 doses of zoledronate at 18-month intervals followed by less frequent dosing could be broadly used for fracture prevention in older postmenopausal women. Having said this, there is no trial directly comparing anti-fracture efficacies between zoledronate at 12- and 18-month intervals in those at high fracture risk, and such a trial is unlikely to be undertaken.

#### Safety

Giving bisphosphonates intravenously bypasses the problem of gastrointestinal intolerance, but about one-third of patients have a flu-like illness (acute phase response) after the first dose of zoledronate (84). Typical symptoms are mild fever, musculoskeletal pain, and malaise that resolve within 2 to 3 days. A few percent of patients report more severe symptoms, and uveitis occurs in 1% of bisphosphonate-naive subjects (85). Uveitis responds quickly to topical corticosteroids, usually under an ophthalmologist's supervision. Symptoms are much less common following second infusions and are seldom observed subsequently. Paracetamol or ibuprofen reduce symptom severity by about one-half (86, 87), and the problem can be effectively eliminated with the use of dexamethasone (4 mg daily for 3 doses from the time of zoledronate infusion) (88).

In contrast to oral bisphosphonate use, there are reports of acute renal failure following zoledronate administration, usually in those with an estimated glomerular filtration rate (eGFR) < 35 mL/min pre-dosing. This eGFR is generally regarded as the cut-off below which this drug should not be used. Using this eGFR threshold (rather than creatinine clearance), two large cohorts have received infusions with acceptable renal safety, though the authors recommend infusion times of 30-60 minutes when eGFR is <50 mL/min (89, 90). Zoledronate doses of 1 or 2.5 mg have good BMD efficacy over 1 year (76), so dose reduction is a further option to improve renal safety in at-risk patients. ONJ and AFF are discussed subsequently.

### Denosumab

Denosumab is a monoclonal antibody directed at the protein RANKL. RANKL is produced in many cells including osteocytes and osteoblasts and is the principal regulator of osteoclastogenesis. As a result, denosumab is a potent antiresorptive agent that lowers bone resorption markers to undetectable levels within days of its administration. Biochemical markers of bone formation fall by >60% in subsequent months. This dramatic suppression of both bone formation and resorption is evident in bone biopsies, with a median bone formation rate of zero after 2 to 3 years of use (91). Unlike the bisphosphonates, denosumab is not selectively taken up into the skeleton, and its duration of action is determined by its persistence in the circulation. It is administered every 6 months by subcutaneous injection. Bone resorption increases steeply in the 7 to 9 months after the last injection, reaching >150% of pretreatment levels at 1 year. Within 18 months of the last dose, all BMD gains have been reversed (92). The increases in bone resorption and the rates of bone loss tend to be greater in those who have received longer treatment with denosumab (93).

#### Efficacy

The pivotal phase 3 trial randomized 7868 postmenopausal women to receive subcutaneous injections of denosumab (60 mg) or placebo every 6 months for 3 years (94). Denosumab increased BMD similarly to the changes found previously with zoledronate and also reduced fracture rates—hip fractures by 40%, nonvertebral fractures by 20% and vertebral fractures by 68%. At the end of 3 years, 60% of the denosumab group continued on open-label treatment, 34% of the original group completing follow-up to 10 years (95). Interim publications suggested that nonvertebral fracture rates declined abruptly beyond 3 years, but this could be attributable to the substantial loss of participants at this time. Selective loss of frailer individuals is to be expected in long-term trials, and noncompliant participants were not eligible for the extension. Annual vertebral fracture rates were higher during the trial extension than they had been in years 1 through 3, so the combined (vertebral plus nonvertebral) fracture rate in the extension was comparable to what it had been in those treated with denosumab in the core study (95).

However, total hip BMD did continue to increase during the extension reaching 9% above baseline at 10 years, in contrast to what occurs with bisphosphonates where BMD plateaus at 3 to 4 years. The mechanism of this ongoing BMD gain is not fully explained because there is stable suppression of turnover markers from year 2 onwards, and bone matrix mineralization (assessed by digitized quantitative microradiography of bone biopsies) is maximized after 5 years of denosumab treatment (95).

#### Safety

Denosumab is initially a straightforward medicine to use, seldom causing side effects, and a cohort has been followed to 10 years without apparent adverse effects (95). Hypocalcemia was not more common in the denosumab group than in placebo in the phase 3 study (94), but has been reported postmarketing, mainly in patients with renal impairment (96). In a Danish cohort of patients with CKD and osteoporosis treated with denosumab (mean eGFR 43 mL/min), 21% developed hypocalcemia, 7% were symptomatic, and 4% needed to be hospitalized (97). In a Boston cohort, changes in serum calcium after the initial dose of denosumab were related to renal function (eGFR ≥60, −0.34 mg/dL; eGFR 30-59, −0.52 mg/dL; eGFR <30, −1.12 mg/dL, *P* < .001) and were less marked after subsequent doses (98). In a study of US Medicare dialysis-dependent women aged >65 years initiating denosumab or oral bisphosphonates, the 12-week cumulative incidence of severe hypocalcemia (<7.5 mg/dL or needing acute care) was 41% with denosumab and 2.0% with bisphosphonates (99). Therefore, if denosumab is to be used in those with eGFR <30 mL/min, it is important to ensure they are vitamin D replete, are taking calcium supplements (particularly in the early weeks after denosumab dosing), and that serum calcium is checked about 10 days postdose (98). Because intravenous bisphosphonates are contraindicated in this patient group, denosumab is often used because it does not negatively impact renal function. However, if denosumab subsequently needs to be discontinued, there are few options to prevent rebound bone loss with its associated risk of fractures.

**Figure 3:**
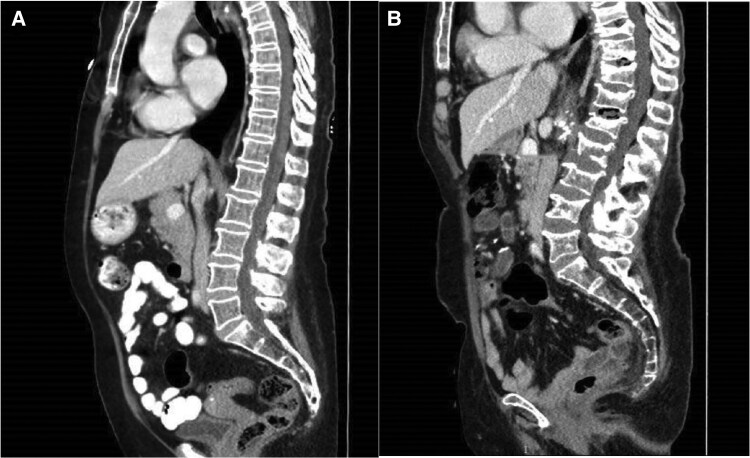
Computed tomography scans of the spine in a 70-year-old woman carried out several months apart demonstrating the occurrence of 9 vertebral fractures following cessation of denosumab after 5 doses. From Tripto-Shkolnik et al, Calcif Tissue Int, 103:44, 2018 (101), used with permission.

The main safety concern with denosumab is its abrupt offset beyond 6 months after an injection. The resulting rapid bone loss can cause clusters of “rebound” vertebral fractures in a few percent of patients, particularly those who have been treated for several years or who have a history of vertebral fractures (100) (Fig. 3). In the denosumab phase 3 trial extension, participants stopping denosumab after >3 years treatment had a rate of multiple vertebral fractures of 7.5/100 patient-years, and the rate of >4 vertebral fractures was 3.3/100 patient-years (102). In those stopping placebo, these rates were 3.6 and 0.6, respectively. Analysis of a national database in Taiwan found the hazard ratio for major osteoporotic fracture in patients discontinuing denosumab after 1-year's use was 1.6 (95% CI, 1.2-2.1) compared with persistent users, and for those discontinuing after 2 years the hazard ratio was 2.6 (1.5-4.6) (103). In contrast, increased fracture risk was not seen in patients discontinuing bisphosphonates. A similar national database study in Korea found that delay of denosumab dosing by 1 to 3 months increased risk of a clinical fracture by 20% and a > 3-month delay almost doubled the fracture risk (104). Therefore, patients considering denosumab treatment need to be aware of its rapid offset before they start it. They must be prepared to adhere to a strict dosing regimen and to transition to an alternative agent if they wish to discontinue denosumab. Recent evidence from North America is that the average length of denosumab use is 2 years, and only 10% of patients transition to another agent (105). The need for such a transition may be lower in the elderly who could reasonably stay on denosumab lifelong, but, because of this potential need for transition, denosumab is not ideal in younger patients with a long life expectancy. Denosumab's safety to 10 years is established by the phase 3 extension study and by postmarketing clinical experience. There is no clear evidence that denosumab should be discontinued after 10 years, although sustaining such marked reduction in bone turnover indefinitely remains a theoretical concern.

The usual agent to transition to is a bisphosphonate. Results are variable (106), but most find that changing to zoledronate or alendronate is associated with some BMD loss in year 1 and with stability thereafter (107-110). Raloxifene or risedronate are less effective (109, 111). The increase in bone resorption after discontinuation is more vigorous after >3 years of denosumab use, so the posttransition BMD loss tends to be greater. Although denosumab does increase BMD more than zoledronate, there is no clear evidence that denosumab has superior antifracture efficacy to bisphosphonates (see Fig. 1), so starting denosumab with the expectation of a future transition to a bisphosphonate is difficult to justify. Such patients could be managed satisfactorily with a bisphosphonate from the outset (see discussion of goal-directed therapy under *Monitoring Treatment Response*). Bisphosphonate intolerance or renal impairment are common reasons for choosing denosumab, so later transitioning to a bisphosphonate might not be an option. These issues should be discussed with patients before denosumab is initiated. Other postdenosumab transitions that have been examined are to romosozumab, which stabilized BMD in a small cohort who had received only 1 year of denosumab (112), and to teriparatide, which is associated with BMD loss at the hip and radius similar to that occurring without intervention, so should not be used (113). Neither of these agents can be used long-term so they do not really address the problem.

### Estrogen agonists and selective estrogen receptor agonists

Estrogen receptor agonists, including estrogen and selective estrogen receptor agonists (SERMs), reduce bone resorption. In the case of estrogen, this results in reductions in vertebral, hip, and total fractures comparable to other antiresorptive drugs (114). Before the advent of potent bisphosphonates, estrogen had a central place in osteoporosis management. However, the many other effects of estrogen (eg, on thromboembolism, cardiovascular disease, and cancer) have complicated its use considerably, so it has not been recommended as a primary agent in fracture prevention in recent years. The risk-benefit analysis for estrogen appears to be more positive in women in their first decade postmenopause (115, 116), so it may have an important role in those with elevated fracture risk in their 50s. There is also an ongoing reappraisal of the safety of hormone treatment in women older than age 60 years in the light of longer term data from the Women's Health Initiative and other studies, and the availability of transdermal estrogen and newer progestin preparations, and expert opinion is becoming more positive about its use (117). It is likely that large trials examining the risk-benefit profile of these newer preparations in the targeted populations will be needed to change current recommendations regarding fracture prevention.

Tibolone has mixed estrogenic, progestogenic, and androgenic effects. It reduces risk of vertebral and nonvertebral fractures (118) but it increases stroke risk. Data on breast cancer risk are mixed. It is available in Europe for management of osteoporosis and menopausal symptoms.

Raloxifene has been the most widely used SERM for osteoporosis. It has lesser effects on BMD than most bisphosphonates and reduces risk of vertebral but not nonvertebral fractures (119). It is associated with a relative risk of thromboembolic disease of 3.1 (95% CI, 1.5-6.2) and of breast cancer of 0.3 (0.2-0.6). Bazedoxifene has similar bone effects to raloxifene (120). Lasofoxifene was associated with reduced risks of nonvertebral and vertebral fractures, estrogen receptor-positive breast cancer, coronary heart disease, and stroke but an increased risk of venous thromboembolic events (121). However, it is not widely available.

### Rare adverse events

AFF and ONJ have emerged as key, long-term safety issues associated with antiresorptive drugs. Table 2 sets out representative incidences of these 2 problems in patients using these agents.

**Table 2:** Incidence of osteonecrosis of the jaw and atypical femoral fractures with long-term use antiresorptive drugs for osteoporosis

	AFF	ONJ
Oral bisphosphonate	6.0 (after 5-8 years use)	∼5 (after 5-10 years)
Zoledronate	∼0	0.6
Denosumab	0.8 (after 4-10 years use)	5.2 (after 4-10 years use)
Non-BP users	0.1	0.8, 1.1, 6.9*^a^*

Data are events per 10 000 person-years.

Abbreviations: AFF, atypical femoral fracture; ONJ, osteonecrosis of the jaw.

^*a*^The 3 figures for ONJ incidence in nonbisphosphonate users are: 0.8 in the placebo group from the Black zoledronate trial, cases adjudicated (71); 1.1 in the Danish cohort of alendronate users in the year before they started alendronate, cases identified from surgical database codes (122); 6.9 in women with osteoporosis from the Taiwan National Health Insurance Research Database treated with nonbisphosphonate drugs, cases identified from database codes and temporally associated antibiotic use (123).

AFF data for oral bisphosphonate users and for non-BP users are from the Black et al study in Southern California (124). Zoledronate data for AFF are from Black et al trial extensions (72, 73), Reid et al 2018 (77) and extension (80), and Bolland et al (48), together comprising 17 930 person-years of follow-up. ONJ data for zoledronate are from these studies plus Black et al (71), Lyles et al (74) and McClung et al (81), comprising 31 333 person-years of follow-up. Denosumab data for ONJ and AFF are from the FREEDOM trial extension (95). Oral bisphosphonate ONJ data are for surgical cases only, from Denmark (122).

#### Atypical femoral fractures

In 2007 and 2008, a group of orthopedic surgeons from Singapore published a case series of 17 women with low-energy, subtrochanteric fractures presenting over a 20-month period (125, 126). All were treated with alendronate. The authors concluded that these were insufficiency stress fractures, characterized by lateral cortical thickening and a predominantly transverse fracture line with a medial cortical spike. Most patients had prodromal pain, and many had bilateral stress reactions or fractures. Similar studies from elsewhere followed (127, 128) and reinforced an earlier series that suggested that oversuppression of bone turnover might increase skeletal fragility (129). These are now referred to as atypical femoral fractures and have formal diagnostic criteria (130). Their etiology is uncertain, but they are not simply attributable to severe suppression of bone turnover because they are principally seen in patients taking oral bisphosphonates long-term, which are less potent antiresorptive agents than denosumab or zoledronate. The focal uptake of bisphosphonates into spontaneous femoral stress fractures could impair healing of these lesions, leading to their progression to complete fractures (131).

The epidemiology of AFFs has recently been described in a cohort of almost 200 000 bisphosphonate users, 99.3% taking oral agents, drawn from the Southern California Kaiser Permanente database (124). From an incidence of 0.1 per 10 000 patient-years in nonusers of bisphosphonates, rates increased to 2.5 in years 3 through 5, and to 13 per 10 000 patient-years with >8 years of use. AFF risk was not related to BMD. A nationwide study in Denmark yielded similar findings, also reporting that glucocorticoid and proton pump inhibitor use were independently associated with increased AFF risk, and that 27% of AFF patients had never used antiresorptive medication (132). The documentation of AFFs in individuals never having received antiresorptive therapy is important because it means that causal inference requires careful epidemiology studies and not just a single case or small uncontrolled series.

The Californian study also addressed effects of ethnicity (124). AFF rates were 5-times higher in Asians compared to Whites, but in both groups substantially more clinical fractures were prevented by bisphosphonates over 5 years use than the number of AFFs occurring (Whites, 107 fractures prevented per AFF; Asians, 14 fractures prevented per AFF). This ethnic difference in risk might be related to differences in femoral geometry (131).

In the Californian study, AFF rates decreased sharply after bisphosphonate discontinuation, reaching near-baseline levels after 15 months off-treatment (124). This suggests that a “drug holiday” could substantially mitigate risk. A similar posttreatment fall in AFF rates was seen in the Danish study. BMD loss after cessation of alendronate varies between studies, being present after 1 year in some studies (61, 133, 134), but BMD gains persisting to 2 years in others (60). The risk of osteoporotic fractures does not increase until after this time (135, 136). Therefore, a 1- to 2-year drug holiday is recommended after 5 years of alendronate use, and this is particularly important in Asian patients because of their higher risk. However, a 6- to 12-month holiday might be more appropriate with risedronate because its antiresorptive effect is lost within 6 months of drug withdrawal and fracture rates rise when it is stopped for >1 year (62). Patients who still have an elevated risk of fracture should recommence treatment at the conclusion of their drug holiday.

There is scant evidence that zoledronate increases AFF risk when dosed at intervals of ≥12 months for osteoporosis, though it does increase risk when dosed monthly in patients with cancer (137). AFFs were actively searched for and none found in the phase 3 trial extension (73), in our 6-year 2000-woman trial and its extension to 10 years (77, 80), and in our recent 10-year 1000-woman trial (48). Collectively, this represents 17 930 person-years of observation. Furthermore, the Swedish national database of AFFs shows no such fractures in those who have used zoledronate only (Karl Michaelsson, personal communication, May 2025). This lower risk is to be expected since currently used interdose intervals with zoledronate are comparable to or greater than the drug holidays that have been associated with dramatic reduction in AFF risk (124). Zoledronate administration at 18-month intervals to postmenopausal women results in bone turnover marker levels at about the premenopausal mean (77, 138), indicating a restoration of normal premenopausal physiology without oversuppression.

There were no AFFs in the core denosumab 3-year study, but 2 occurred during the extension, a rate of 0.8 per 10 000 patient-years (95). This rate is well below that found in oral bisphosphonate users after a similar period of treatment in the Southern Californian cohort (∼6 per 10 000 patient-years), but probably higher than with zoledronate (see Table 2). A case series has suggested that denosumab use following bisphosphonate has a higher risk than either agent alone (139).

#### Osteonecrosis of the jaw

Necrotic lesions in the mouth were reported in the early 2000s following the advent of monthly parenteral treatment with intravenous bisphosphonates for the prevention of skeletal-related events in patients with advanced cancer (140). This report also included a small number of patients receiving oral bisphosphonates for osteoporosis. Similar lesions were reported when denosumab was administered monthly to patients with advanced cancer (141). An ONJ lesion is often precipitated by an extraction or other invasive dental procedure in at-risk individuals. ONJ was subsequently defined as the presence of exposed bone in the mouth persisting for >8 weeks despite appropriate treatment, though dentists sometimes diagnose “stage 0 ONJ” in patients receiving antiresorptive therapy who have only nonspecific oral findings and no exposed bone in the mouth (142). This practice substantially impacts on ONJ incidence and requires cautious interpretation of any nonadjudicated case series. Estimates from adjudicated case series of the incidence of ONJ in patients taking oral bisphosphonates are mostly of the order of 1 in 100 000, with no cases reported from the principal clinical trials of these drugs despite accumulating >60 000 patient-years of exposure to bisphosphonate treatment (143). In contrast, a recent analysis of the United Kingdom Clinical Practice Research Datalink, which included “manual electronic record review,” found a incidence rate of 1.1 per 10 000 patient-years in current bisphosphonate users compared with a rate of 0.8/10 000 patient-years in past users of any osteoporosis drug (144). Incidence rates were much lower in alendronate users with the diagnosis of osteopenia (0.2) as opposed to osteoporosis (3.2), suggesting an effect of underlying bone health. The cumulative incidence of ONJ was 5/10 000 patient-years after 5 years and 18/10 000 patient-years after 10 years. ONJ incidence dropped to near zero within 9 months of drug discontinuation, such an abrupt change suggesting that ONJ was being selectively diagnosed in bisphosphonate users. These incidence rates are broadly similar to those reported in a nationwide study of surgically treated ONJ from Denmark in 2017 (122). A broad range of chronic illnesses were associated with increased ONJ risk in both these studies.

In the zoledronate clinical research program in osteoporosis, where a specific expert group adjudicated all dental adverse events, there was 1 case in the zoledronate group and one in the placebo group, generating a combined incidence of <1 per 14 200 patient-years in both groups. We have seen no cases in our two 10-year zoledronate studies (48, 77, 80). Subsequent database studies of oral bisphosphonate users have usually not adjudicated events and have sometimes assessed categories of dental surgeries rather than the presence of exposed bone in the mouth. As a result, incidences vary widely (see Table 2), sometimes being similar among patients with osteoporosis whether or not they received bisphosphonates (123, 145, 146), whereas other studies indicate greater risk in those with longer use and higher compliance (122). As a result, and because of the lack of evidence of ONJ in trials of antiresorptive drugs in osteoporosis, doubt remains regarding the causative role of osteoporosis doses of these drugs in ONJ. Other risk factors for ONJ appear to be smoking, glucocorticoid therapy, chemotherapy, diabetes, existing dental inflammation, and poor dental hygiene (147). Case series in osteoporotic patients indicate these ONJ-like lesions are of lower grade than those seen in patients with cancer (148) and that they are more likely to heal (149). The rarity of ONJ in osteoporosis patients means that routine dental examinations before starting antiresorptive treatment are not required (147), in contrast to current oncology practice. One small, randomized trial has demonstrated increased rates of healing of ONJ in patients treated with teriparatide for 8 weeks (150). The American Association of Oral and Maxillofacial Surgeons has recently updated its position paper on ONJ, which discusses management of this problem (151).

## Anabolic agents

During the 1990s, even as ever more powerful antiresorptive agents were developed, there remained a desire to address the other half of the bone balance equation—that is, to produce an effective osteoblast stimulating agent. PTH is a physiological stimulator of osteoblast activity that, because of the coupling of the activities of osteoblasts and osteoclasts, also stimulates bone resorption. Although bone resorption is dominant in primary hyperparathyroidism, daily pulse of PTH was found to have beneficial effects on BMD in trials of up to 3 years (152). This led to a phase 3 trial of human PTH (1-34), now known as teriparatide.

### Teriparatide

The phase 3 study randomized 1637 postmenopausal women with vertebral fractures to 3 groups: placebo, teriparatide 20 µg, and teriparatide 40 µg daily by subcutaneous injection (153). The study was terminated after a mean duration of 18 months, following the demonstration of osteosarcomas in rat studies being run concurrently. Vertebral fracture risk was reduced by 65% in the 20-µg group, although there was no effect on height loss. Nonvertebral fracture risk was reduced by 35% in the 20-µg group, although there were relatively few women with fragility fractures (58) or hip fractures (9) in the trial. At study end, lumbar spine BMD increased 9.7% and total hip 2.6% above baseline in the 20-µg group. BMD data during the first year of the study are not presented. The radial shaft BMD decreased by 2.1% in the 20-µg group and by 3.2% from baseline to study end. Subsequent studies have shown stable or falling hip BMD in the first year of teriparatide use (113, 154) (though most of these patients had previously been oral bisphosphonate users) and decreases in radius BMD over 2 years (113).

There are scant data addressing the prevention of hip fractures with teriparatide. A meta-analysis of 23 studies employing a variety of comparators, identified only 34 incident hip fractures among 8644 women and men, but did suggest prevention of hip fracture by teriparatide (odds ratio 0.44 [95% CI, 0.22-0.87] (155). The effects on the risk of humerus (1.02 [0.50-2.08]), forearm (0.53 [0.26-1.08]), and wrist fractures (1.21 [0.72-2.04]) were not statistically significant in this analysis. A recent network meta-analysis did not demonstrate that teriparatide reduced hip fractures more or less than other agents or placebo (156), possibly reflecting the lack of data and, thus, statistical power. Teriparatide effects on BMD over 1 year in men are similar to those seen in women, but antifracture efficacy in men has not been established (157). In a randomized 18-month study of teriparatide or alendronate in glucocorticoid-induced osteoporosis (n = 428) (158), there were fewer vertebral fractures in the teriparatide group than in the alendronate group (0.6% vs 6.1%, *P* = .004), but the incidence of nonvertebral fractures was similar (5.6% vs 3.7%, *P* = .36).

The antifracture efficacy of teriparatide compared with an oral bisphosphonate was assessed by the VERO study, a 2-year trial comparing teriparatide with risedronate in 1360 women with prevalent vertebral fractures (159). The primary outcome was new radiographic vertebral fractures. The risk ratio for vertebral fractures was 0.44 (95% CI, 0.29-0.68) and the hazard ratio for nonvertebral fractures was 0.70 (0.46-1.05). There were 2 and 6 hip fractures in the teriparatide and risedronate groups, respectively. A recent comprehensive network meta-analysis comparing treatment using a PTH receptor agonist with use of a bisphosphonate found a relative risk of any clinical fracture of 0.61 (95% CI, 0.39-0.94) (160). PTH receptor agonists were also superior to bisphosphonates for prevention of vertebral fractures, as found in the VERO trial.

The most widely used dose of teriparatide is 20 µg/day. In the phase 3 trial, 40 µg/day also prevented fractures but caused more adverse events. In Japan, a trial comparing weekly teriparatide (56.5 µg) with placebo in 578 women and men over 72 weeks, found a relative risk of vertebral fracture of 0.20 (95% CI, 0.09-0.45) and of nonvertebral fracture of 0.98 (0.47-2.07) (161). A further study compared this weekly regimen with alendronate in 1011 women over 72 weeks. The rate ratio for vertebral fractures was 0.78 (0.61-0.99) and for nonvertebral fractures was 1.09 (0.68-1.75). Together, the higher adverse events with 40 µg daily and the lack of nonvertebral fracture efficacy in weekly administration suggests that the optimum dosing, based on current evidence, is 20 µg daily.

#### Safety

Teriparatide doses >20 µg/day can be associated with headaches and nausea (153, 161). Mild hypercalcemia sometimes occurs even on 20 µg/day and responds to adjusting dose frequency or eliminating calcium supplements if being used. Occasionally, patients on teriparatide complain of limb pain, though this was not more common in the teriparatide trials. It also responds to dose frequency adjustment. The principal safety concern with teriparatide was the finding in rat studies that long-term, high-dose use of teriparatide increased the risk of osteosarcoma. As a result, monitoring programs for this malignancy were established in teriparatide users and have not demonstrated that this is a problem in humans over 15 years of follow-up (162, 163). Accordingly, the Food and Drug Administration (FDA) removed its “black box” warning and now permits use for >2 years in those at continuing high risk of fracture.

### Abaloparatide

Abaloparatide is a synthetic, 34-amino acid peptide that shares 41% homology with PTH (1-34) and 76% homology with PTH-related peptide (1-34) (164). Like PTH, it activates the PTH1 receptor signaling pathway, but was developed with a view to promoting the more osteoanabolic conformation of that receptor (165).

An 18-month, phase 3 study randomized 2463 osteoporotic postmenopausal women to placebo, abaloparatide 80 µg/day, or open-label teriparatide 20 µg/day (166). Abaloparatide increased BMD more than teriparatide, and both agents reduced vertebral fractures by ≥80% compared with placebo. Using the full nonvertebral fracture database, the hazard ratio for abaloparatide was 0.57 (95% CI, 0.32-1.00), and for teriparatide was 0.72 (0.42-1.22). A study extension was run for a further 2 years, during which women from the abaloparatide and placebo groups took alendronate (167). Over the entire trial period, comparisons between the abaloparatide/alendronate and placebo/alendronate groups found a 84% reduction of vertebral fractures (*P* < .001) and a 39% reduction for nonvertebral fractures (*P* = .04). In the abaloparatide group, there were more discontinuations secondary to adverse events, mainly related to nausea, dizziness, headache, and palpitations.

Abaloparatide was approved in the United States in 2017 but in Europe this took longer. Initial applications were declined because the data on prevention of nonvertebral fractures were considered to be weak after 2 clinical sites were deleted from the database as a result of Good Clinical Practice shortcomings, and because there was concern regarding tachycardia after dosing. An extension of the original phase 3 study and postmarketing safety data were used to address these issues, leading to its approval in Europe in December 2022 (164, 168).

Subsequently, a study of an administrative claims database found that abaloparatide and teriparatide had comparable effects on cardiovascular safety (169). Near lifelong treatment of rats with abaloparatide resulted in dose- and time-dependent development of osteosarcomas, comparable to the response to hPTH (1-34) at similar exposure (170). Accordingly, abaloparatide use is limited to 2 years.

## Dual-acting agent—romozosumab

Romosozumab is a humanized, monoclonal antibody directed at sclerostin. Osteocytes release sclerostin at the surface of bone, where it inhibits Wnt signaling in osteoblasts and also down-regulates expression of osteoprotegerin (171). As a result, sclerostin blockade both stimulates bone formation and inhibits bone resorption, hence its designation as a “dual-acting agent.” Romosozumab is administered by monthly subcutaneous injection over a period of 12 months.

### Efficacy

The phase 2 study demonstrated that romosozumab 210 mg/month doubled bone formation markers and reduced resorption markers by 30% in the first month (172). Both formation and resorption markers returned to near baseline levels by 3 months, but increases in BMD were ongoing to 2 years, reaching 11% at 1 year and 15% at 2 years in the lumbar spine (172, 173). The BMD effects in the first year were substantially greater on both spine and hip BMD with romosozumab than with either alendronate or teriparatide. The effects on both spine and hip BMD were substantially reversed following romosozumab withdrawal (173, 174) but BMD was regained after reinstating romosozumab treatment for a further year (174). Transitioning from romosozumab to either denosumab (174) or zoledronate (175) maintained the increases in BMD achieved with romosozumab. The sequence romosozumab-denosumab-romosozumab maintained a gentle upward trend in BMD, but the second romosozumab treatment did not reproduce the substantial step-up in BMD seen when romosozumab was first introduced (174). Bone biopsies show substantial increases in bone formation rates with reduction in numbers of osteoclasts, indicating that dissociation of these 2 components of bone turnover has been achieved (176). Interestingly, in the phase 3 FRAME trial, bone resorption markers remained suppressed through to 12 months, consistent with these histology findings (177). Men treated with romosozumab for 1 year showed similar changes in BMD (178).

The romosozumab phase 3 program involved 2 sequential trials. In FRAME, 7180 osteoporotic women were randomized to monthly romosozumab or placebo for 12 months, then both groups were switched to denosumab (177). Romosozumab reduced vertebral fractures by 73% (*P* < .001) and nonvertebral fractures by 25% (*P* = .1) at 1 year, with similar results at 2 years. In a study extension for a further year, denosumab was continued in both groups (179). Over the whole 3 years, vertebral fractures were reduced by 66% (*P* < .001) and nonvertebral fractures were reduced by 21% (*P* = .04) in the romosozumab-denosumab group compared with the placebo-denosumab group.

The second part of the phase 3 platform was ARCH. Osteoporotic women (N = 4093) were randomized to weekly oral alendronate or monthly romosozumab in year 1, then all participants took alendronate alone for up to 48 months. The primary end points were the cumulative incidence of new vertebral fracture at 24 months and the cumulative incidence of clinical fractures (nonvertebral and symptomatic vertebral fracture) at the time of the primary analysis (after clinical fractures had been adjudicated in ≥330 patients). At 24 months, the risk of vertebral fractures was 48% lower in the romosozumab-alendronate group compared with alendronate alone (*P* < .001). The primary analysis was undertaken at a median of 2.7 years and showed that clinical fractures were reduced by 27% (*P* < .001), nonvertebral fractures by 19% (*P* = .04), and hip fractures by 38% (*P* = .02) (180).

### Safety

Adverse events were generally balanced between the groups, though some women experienced mild injection site reactions from romosozumab. In FRAME, 1 atypical femoral fracture and 2 cases of ONJ were observed in the romosozumab group only (177). In ARCH, there were no cases of ONJ or AFF in year 1. Subsequently, there was 1 case of ONJ in each group, and 6 AFFs—2 in the romosozumab-alendronate group and 4 in alendronate-only group (180).

A critical safety issue with romosozumab is the imbalance in adjudicated, serious cardiovascular events observed in year 1 of ARCH (2.5% in romosozumab group vs 1.9% with alendronate (odds ratio 1.31 [95% CI, 0.85-2.00]) (180) but absent from FRAME. The excess was principally in cerebrovascular events and cardiac ischemic events. The reason for the inconsistency between the 2 studies is uncertain. It could be a chance finding, as there were few women (<25) with cardiac or cerebrovascular events, and overall there was a difference of only 12 women between the groups of >2000 women. Numerous adverse events were assessed in the study, and it is to be expected that some events, especially when the numbers are sparse, will differ between groups. That view is reinforced by the absence of a similar signal in FRAME. It could also represent a cardioprotective effect of the bisphosphonate, which has been found in some other studies (77) but certainly not consistently across this drug class. Animal studies have not found adverse cardiovascular effects of romosozumab (181). However, sclerostin is expressed in the heart, aorta, coronary, and peripheral arteries, and expression is increased at sites of arterial calcification in rodents. Animal studies suggest a cardiovascular protective role of sclerostin but findings have been inconsistent (182). As a result of these findings, the FDA has issued a black box warning indicating that romosozumab may increase the risk of myocardial infarction, stroke, and cardiovascular death, and that it is contraindicated in patients who have had a myocardial infarction or stroke within the past year.

## Calcium supplements

Calcium, along with phosphate, forms the mineral component of bone, providing the compressive strength and rigidity characteristic of this tissue. However, the fundamental structure of bone consists of type I collagen, laid down by osteoblasts and remodeled by osteoclasts. This structure becomes mineralized if extracellular fluid concentrations of calcium and phosphate are in the normal range. Reductions in circulating concentrations of both ions do occur in severe vitamin D deficiency, causing rickets/osteomalacia. Very low calcium intakes (<200 mg/day) alone have been reported to cause hypocalcemia and osteomalacia in African children (183), though intakes <300 mg/day have been common in Africa and Asia and not associated with bone pathology or increased fracture risk in adults (184, 185).

**Figure 4:**
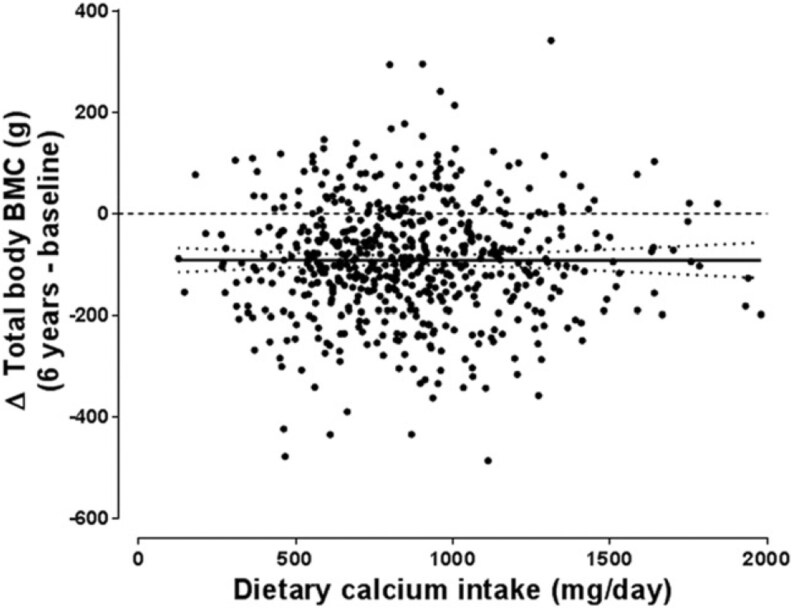
Absolute change (Δ) in total body bone mineral content (BMC) over 6 years in 698 osteopenic postmenopausal women not receiving bone-active medications, in relation to each woman's average calcium intake assessed at baseline, year 3, and year 6. The regression line (with 95% CIs) for this relationship is shown (*P* = .99). From Bristow et al, J Clin Endocrinol Metab, 104:3576-3584, 2019 (191), used with permission.

Calcium balance studies have been used to determine the optimum calcium intake and led to the Food and Agriculture Organization and the WHO in 1974 recommending minimum intakes of calcium for adults of 400 to 500 mg/day (186). Several years later, Heaney showed that calcium balance was directly related to calcium intake and that extrapolation of the data indicated that zero calcium balance could be achieved in postmenopausal women at an intake of 1500 mg/day (187, 188). It is likely that these conclusions were incorrect as a result of the short-term nature of balance studies (189) and because of statistical flaws in their analysis. Longitudinal BMD studies have not demonstrated a dependence of bone loss on stable calcium intakes across a range from 300 to 2000 mg/day (190-192). More recent balance studies have not confirmed the need for such high intakes, suggesting that 300 to 400 mg/day is adequate (193, 194). In fact, the advent of whole-body dual-energy X-ray absorptiometry (DXA) scanning has rendered calcium balance studies obsolete for assessment of optimal calcium intake because this DXA technique directly measures *bone* balance over periods of years. DXA-measured bone balance in postmenopausal women and in men is independent of calcium intake over the 300 to 2000 mg/day range (190, 191, 195) (Fig. 4). The NHANES study found no relationship between calcium intake and BMD in adults aged >50 years (196) and meta-analyses of observational studies show that fracture risk is independent of calcium intake (197, 198). Hip fracture incidence varies widely between countries and does show evidence of tending to be lower in those countries with high calcium intakes (185). In the analyses that led to the development of the principal fracture risk calculators (FRAX and Garvan), calcium intake was not predictive of fracture so is not used in these instruments.

Bone densitometry has permitted randomized trials of the effects of calcium supplements on BMD. In premenopausal women, there are not clinically significant effects on BMD (199). However, calcium supplements do have small positive effects on BMD in older women, producing between-groups differences of about 1% in total hip BMD in the first year of study but not increasing subsequently (200). Recent meta-regression analyses of osteoporosis trials by Black et al (201) show that a change in total hip BMD of 2.1% at 2 years is required for prevention of nonvertebral fractures and of 3.2% for prevention of hip fractures, suggesting that fracture prevention from calcium supplements is unlikely. This is what most clinical trials have now confirmed, with a comprehensive meta-analysis of trials concluding that “the use of supplements that included calcium, vitamin D, or both compared with placebo or no treatment was not associated with a lower risk of fractures among community-dwelling older adults” (202). The SWAN study, a longitudinal study of perimenopausal women, also found that calcium supplement use was associated with small benefits on BMD but did not affect fracture rates (203). These findings are reflected in recommendations of the US Preventive Services Task Force (204, 205) and the International Osteoporosis Foundation (206) discouraging calcium supplement use in the community for fracture prevention. It should be noted that these conclusions relate to community-dwelling adults. As noted previously, the Chapuy study of vitamin D plus calcium in vitamin D-deficient women in nursing homes, did find a reduction in fractures (39, 40), probably because it was a carried out in a cohort of frail elderly women with severe vitamin D deficiency, of whom many probably had unrecognized osteomalacia.

With recent studies having failed to find any significant benefit from the use of calcium supplements, it is also important to remember that these supplements do have significant side effects, particularly gastrointestinal (eg, bloating, constipation). In clinical trials of calcium, gastrointestinal symptoms are increased by 43% compared with placebo, and hospital admissions for acute abdominal problems are doubled (207). In the Women's Health Initiative trial of calcium and vitamin D, the risk of renal calculi was increased by 17% (42), and hypercalcemia and hypercalciuria are common (9% and 31%, respectively) (208). Meta-analyses of randomized trials have reported that myocardial infarction risk is increased by calcium supplement use with or without vitamin D (209, 210). This effect might be mediated by the abrupt increase in serum calcium that follows ingestion of a calcium supplement that persists for >8 hours (211). The increase in circulating calcium concentration is associated with adverse effects on blood pressure, blood coagulability, and calcification propensity (212-214). There is now abundant evidence from epidemiological (215) and Mendelian randomization (216-218) studies that increases in circulating calcium levels of the magnitude observed after calcium supplements are associated with increased cardiovascular risk. Multiple meta-analyses of calcium supplementation trials demonstrate an adverse trend in incidence of cardiovascular events, with statistical significance varying according to numbers of patients included. Thus, for calcium supplements and myocardial infarction, Bolland et al reported a relative risk of 1.27 [95% CI, 1.01-1.59] (209), Lewis et al a risk ratio of 1.37 [0.98-1.92] (219), Mao et al an odds ratio of 1.28 [0.97-1.68] (220), and Yang et al a relative risk for coronary heart disease of 1.20 [1.08-1.33] (221). None of these findings is reassuring, though some are underpowered as a result of having included fewer studies.

Although the evidence indicates that the use of calcium and/or vitamin D supplements in the community do not prevent fractures, these are still often given to patients receiving pharmaceuticals for osteoporosis because they were used in the pivotal trials of these medications. This is probably unnecessary for most agents, and calcium is likely to cause side effects, thus impacting on adherence to the primary pharmaceutical. A trial directly addressing the additive benefit of calcium to alendronate found that addition of supplemental calcium had no effect on BMD changes, though fractures were not a study endpoint (222). For other agents, direct comparisons of adding calcium have not been performed, but estrogen (49, 223), clodronate (47), and zoledronate (48, 77) without calcium supplementation have demonstrated comparable antifracture efficacy to that achieved with its coadministration. However, it is important to remember that potent antiresorptive agents can cause hypocalcemia if patients are severely vitamin D-deficient: vitamin D supplementation is most appropriate to prevent this problem. Hypocalcemia is also common when denosumab is given to patients with renal impairment (224), and calcium supplements are often needed to mitigate this. The use of anabolic antiosteoporosis medications without calcium supplementation has not yet been systematically assessed.

## When to prescribe antiosteoporosis medications

### Prevention

Over the past 50 years, 2 quite distinct approaches to the use of AOMs in postmenopausal women have been explored—prevention of menopausal bone loss vs treatment of established disease. There was early success with the prevention approach using estrogen, with the demonstration of reduced rates of vertebral fractures in a randomized trial (49). As a result, osteoporosis prevention was among the benefits that contributed to the widespread use of hormone treatment until the publication of the Women's Health Initiative in 2002. This study found a more complex risk-benefit picture, though it did confirm that estrogen prevented postmenopausal bone loss and fractures (114). Calcium and vitamin D were also used for osteoporosis prevention but had little or no effect on BMD and did not prevent fractures. A range of antiresorptive drugs were demonstrated to prevent bone loss in nonosteoporotic women (225, 226), but fracture prevention in early postmenopausal women has only been demonstrated very recently, using zoledronate (48). It is now important to determine how widely this intervention should be deployed in younger postmenopausal women for cost-effective fracture prevention. It is also possible that other antiresorptive regimens that can produce similar long-term stability of BMD might have similar antifracture efficacy, but this needs to be explored further.

Since validated fracture calculators, such as FRAX and Garvan, became available in 2007 (227, 228), the convention has been to base treatment decisions on the 10-year risk of fracture. Cost-effectiveness will be lower in early postmenopausal women because their fracture risk is lower, though in the Bolland zoledronate study (48) the number needed to treat (NNT) for any fracture was 9 for the 5-yearly dose group and 13 for the 1-dose group, suggesting that this regimen is cost-effective, particularly in the group given only 1 zoledronate infusion in 10 years. Another risk parameter to guide decisions for intervention in a prevention scenario is the remaining lifetime risk of fracture. White women aged 50 years with femoral neck T-scores of +1, 0, or −1 have remaining lifetime risks of hip fracture of 10%, 20%, and 40%, respectively (229). This suggests that intervention in 50-year-olds with hip T-score ≥1 is unlikely to be worthwhile, whereas in those with lower BMDs it may well be. Indeed, postmenopausal women in their 50s or 60s with osteopenic BMD seem very appropriate recipients for infrequent zoledronate infusions for long-term fracture prevention.

### Treatment

The second treatment approach to AOM use has been to intervene in those who have already fractured or who are at high risk of fracture, based on low BMD or other risk factors. All the agents currently licensed for treating osteoporosis have been shown to reduce fracture rates in trial cohorts meeting various criteria for osteoporosis. Our 2018 trial of zoledronate in osteopenic women aged older than 65 years sat between the classic prevention and treatment scenarios, in that the participants had already undergone considerable postmenopausal bone loss but did not meet the BMD criterion for osteoporosis. This study demonstrated significant fracture prevention in this osteopenic cohort (77). Other AOMs have also been found to prevent fractures in non-osteoporotic women (230), including clodronate (47), estrogen (49, 114), denosumab (231), and raloxifene (232). Furthermore, large meta-analyses of AOMs have shown that anti-fracture efficacy is independent of baseline BMD (160, 233). Thus, current AOMs can reduce fracture risk across a broad range of those at risk.

The 2018 zoledronate trial in osteopenic women (77) also highlighted the lack of usefulness of the term “osteopenia” in clinical settings. In the trial, the range of estimated 10-year MOF risk was 4% to 50% despite all of them having osteopenia. Thus, many older women with osteopenia have higher fracture risks than younger women with osteoporosis by BMD criteria.

There has been a proliferation of indications for using AOMs for fracture prevention, most based on clinical judgment rather than quantitative analysis. These include a particular BMD (typically a T-score ≤ −2.5), history of fracture, or an elevated fracture risk, the Bone Health and Osteoporosis Foundation defining this as 10-year hip fracture risk ≥3% or MOF risk ≥20% (234). Because estimated fracture risk provides a single parameter that can integrate clinical risk factors and BMD, it is logical to use this as the guiding indicator for recommending treatment. The challenge then becomes to determine at what level of risk, intervention is appropriate.

We have recently advocated that 3 principles should underpin the decision to offer an osteoporosis treatment to an individual (230):

- that the regimen proposed has been proven to prevent fractures in a randomized controlled trial (or this is highly likely based on other trials with similar agents)
- that the intervention is cost-effective in this individual, based on their fracture risk and on the cost of the intervention in that jurisdiction
- that the intervention has an acceptable safety profile.

The first principle is met for all commonly used AOMs in patients with T-scores < −2.5 or with fractures, and for bisphosphonates, estrogen, and probably denosumab in osteopenic individuals. Safety is discussed previously. Oral bisphosphonates have a good safety profile out to 5 years, and with drug holidays can probably be safely used over several treatment-holiday cycles. Zoledronate at intervals ≥12 months appears to have very low risks of both AFFs and ONJ, coupled to the convenience of infrequent dosing, so is suitable across a range of fracture risks. Denosumab effects on BMD and fracture show rapid offset after discontinuation with a mounting risk of rebound vertebral fractures after longer term use (102), so is only suitable in those with high fracture risks where continuous treatment is indicated. Similarly, anabolic agents are appropriate for those with high fracture risk for reasons of cost, safety, and intensity of the treatment regimens.

### Cost-effectiveness

Cost-effectiveness is critical for anabolic AOMs and is discussed under those headings. It is also highly relevant in patients at lower fracture risk considering use of generic bisphosphonates. Indeed, the 3% hip and 20% MOF risk thresholds that are widely used, were derived from cost-effectiveness analyses based on 2008 prices, at which time oral bisphosphonates typically cost US$600/year (235). Because many effective AOMs are now generic and available for a few percent of that cost, these thresholds are no longer valid. The UK National Institute for Health and Care Excellence (NICE) has estimated that oral bisphosphonates are cost-effective at a 10-year probability of osteoporotic fragility fracture of ≥1% (236), which would permit use across almost the entire postmenopausal population. In this context, medication safety and patient motivation become the critical factors in decision making. NICE judged zoledronate to be cost-effective when osteoporotic fracture risk was 10% (236), although this analysis considered annual treatment and only accounted for medication and not infusion costs. Much higher risks are necessary to justify use of anabolic drugs that can cost up to US$50 000/year. Therefore, a single fracture-risk threshold for use of all AOMs is not appropriate.

Leslie has modeled the use of lower MOF risk thresholds for treatment with AOMs in postmenopausal women, using longitudinal databases from Manitoba, Canada (237), and we have summarized these findings recently (230). In women aged >65 years, intervention at a MOF probability of 10% would result in treatment of 62% of the population, including 83% of those who had a MOF and 92% of those who had a hip fracture during 4.4 years follow-up. They estimate that 5.8 fractures would be prevented per 1000 person-years of treatment. Intervention at a MOF probability of 15% would result in treatment of 33% of the population, including 59% of women who had a MOF and 74% of those who had a hip fracture during follow-up. A total of 9.1 fractures would be prevented per 1000 person-years of treatment. These data indicate that the NNT for 5 years to prevent 1 fracture is 34 at a 10% MOF threshold and 22 with a 15% threshold in this cohort. Because about half of older White women have fractures, these intervention rates seem reasonable in this population. The recent trials showing that fracture prevention extends many years beyond the period of treatment with zoledronate means that the actual NNTs may be much lower than those estimated here, if zoledronate were used as the AOM.

It is of interest to compare these simulations with the findings in our 2018 trial of zoledronate in osteopenic women aged >65 years (77). They had a median 10-year MOF risk of 12% (interquartile range 9.2-15) and hip fracture risk of 2.4 (1.5-3.9). Fracture incidence was significantly decreased in each tertile of fracture risk, with NNTs of 19, 12, and 14 in tertiles 1, 2, and 3, respectively (78). These NNTs are lower than in the Manitoba analyses, probably because the treatment period was a year longer and because morphometric vertebral fractures were also included in the fracture endpoint for the zoledronate study. The NNTs suggest that this zoledronate regimen was cost-effective, particularly in those in the upper 2 tertiles of risk (ie, 10-year hip fracture risk >1.8%, MOF risk >10%). Dosing zoledronate every 18 months reduces costs by one-third compared with the NICE analysis. The extension of this study observed stable antifracture efficacy for about 4 years following the last zoledronate infusion, increasing cost-effectiveness and indicating that efficacy could be maintained long-term with even less frequent infusions (eg, 2-3 yearly) (80). Thus, these results from both Manitoba and New Zealand suggest that MOF risks of 10% to 15% over 10 years could be acceptable indications for treatment with generic bisphosphonates in women aged >65 years. Intervention at such lower fracture risks is heavily dependent on patient motivation, and the infrequency of zoledronate infusions is more likely to maintain this than is weekly tablet dosing. Asian patients have a higher risk of AFFs (124), so should be guided away from long-term oral bisphosphonate use when osteoporotic fracture risk is only moderate.

## Choice of initial treatment

Once the decision to offer treatment has been made, the initial AOM needs to be selected. This will vary from country to country according to what is available and the current reimbursement status of medications. As discussed previously, it will also be influenced by the patient's fracture risk, with more expensive medications only being cost-effective in those at higher risk.

In most situations, the initial agent is a bisphosphonate because they are cheap, effective, and safe. An oral bisphosphonate might be more convenient for some since there is no need to provide infusion facilities, but the infrequent dosing of zoledronate and its lower risk of gastrointestinal side effects will appeal to many. This is particularly so following admission to hospital for a fracture, where there is a growing consensus that zoledronate should be administered before discharge (238). Adequate renal function is required for zoledronate administration. One-third of patients will have an acute phase response after the first dose, but this can be virtually eliminated by a 3-day course of dexamethasone, which we use routinely (88).

Denosumab is sometimes used as first-line therapy though it is currently more expensive, and only suitable when strict adherence to the 6-month dosing interval is to be expected. Its antifracture efficacy is comparable to that of bisphosphonates. Denosumab should only be discontinued with transition to another antiresorptive agent, almost always a bisphosphonate. Unlike zoledronate, it does not exacerbate renal impairment, though the possibility of hypocalcemia needs to be managed when patients with CKD are treated with denosumab.

In patients with a high risk of fracture, using an anabolic agent as the first intervention is now advocated by many. This approach is at the heart of the concept of “goal-directed therapy for osteoporosis, which is discussed in more detail in the next section.” The evidence from the VERO study (comparing risedronate with teriparatide) and from the ARCH study (comparing romosozumab with alendronate) demonstrated that these anabolics have greater antifracture efficacy than oral bisphosphonates. However, studies directly comparing the antifracture effects of anabolics with the most potent antiresorptives (zoledronate and denosumab) have not been undertaken.

Teriparatide has little if any effects on BMD of the proximal femur in the first year of treatment and appendicular BMD may actually decrease in this period (113, 239), so its use as an initial treatment in those at high risk of femoral neck or other cortical fractures may not be ideal. In contrast, romosozumab robustly increases spine and hip BMD, and shows broad antifracture efficacy, so is an attractive first-line intervention in patients at high fracture risk. Trials combining teriparatide with either zoledronate (240) or denosumab (113, 239) have shown positive changes in cortical BMD from the outset and show more positive effects than either agent alone. Formal trials of fracture prevention using combination regimens have not been undertaken, though the small trial of teriparatide plus zoledronate did show fewer fractures in the combination treatment group compared with zoledronate alone (240). The lack of demonstrated antifracture efficacy of combination regimens contributes to the infrequency of their use, together with the added costs and, possibly, higher risk of adverse events. However, providing an anabolic to a patient recently treated with a bisphosphonate is, in effect, combination therapy since there will be a persisting antiresorptive effect of the bisphosphonate.

Despite the higher cost of anabolics, their use can be cost-effective if targeted appropriately—a recent analysis of romosozumab use in 74-year-old Swedish women with a recent MOF found an incremental cost-effectiveness ratio of €33 732, well within that country's reference willingness-to-pay (241). Thus, the cost-effectiveness of expensive osteoporosis medications is country-specific because it is determined by fracture risk, treatment costs, and willingness to pay. Also, these determinations will continually change as cheaper biosimilars become available for teriparatide, and in the future for other agents, and jurisdictions revise their willingness to pay.

## Monitoring treatment response

Once an AOM has been instituted, it is usual to monitor the patient's response. With oral bisphosphonates, it is important to verify that the patient is taking the medication regularly and that they are following the dosing requirements (taken fasting, with water alone, at least 30 minutes before other fluids or food). Some patients show poor biochemical or BMD responses to oral bisphosphonates, even when they appear to be following the dosing instructions. Therefore, confirming that a bone turnover marker (usually procollagen type I N-terminal propeptide or collagen type I C-telopeptide) is suppressed below the midpoint of the premenopausal range at 3 to 6 months of treatment is useful confirmation that the drug is being adequately absorbed (242, 243). Monitoring of bone turnover markers improves medication persistence (244, 245) and is associated with lower fracture rates (246). Failure of antiresorptive drugs to suppress markers below the premenopausal mean value requires improved dosing practices or a change to parenteral medication.

Many doctors assume that BMD measurement is critical for monitoring treatment response. Certainly, it is reassuring to see positive BMD changes in those taking oral agents, so a repeat BMD after 3 to 5 years of treatment is common. In some studies, monitoring BMD during follow-up is associated with fewer fractures (247), and more positive BMD changes during treatment are associated with higher compliance and with lower fracture rates (248). Having said this, a patient's first BMD measurement is their most important because it is tightly correlated with follow-up BMD values on treatment (r = 0.93) and is highly predictive of fracture risk while on treatment (249), particularly in those on parenteral agents in whom the vagaries of absorption are removed. Similarly in individuals not receiving AOMs, a single BMD measurement is predictive of fracture risk over 7 years in men (250) and up to 25 years in women (251). In untreated women in their 60s, hip fracture risk assessed with FRAX doubles every 5 to 6 years, so it is possible to determine when an individual will reach any given threshold for intervention without frequent BMD measurements (15). If BMD decreases significantly while on treatment, treatment adherence should be checked, and a search should be made for underlying conditions contributing to ongoing bone loss. Weight loss often contributes to bone loss in older individuals. Bone loss in the presence of adequate suppression of turnover suggests that the addition or substitution of an anabolic drug should be considered.

The advent of potent anabolic drugs introduced the possibility of being able to achieve substantially greater increases in BMD than had previously been possible. This, together with the possibility that adverse events (eg, malignancy, cardiovascular disease) might result from long-term use of anabolics, suggested that it might be appropriate to set BMD treatment targets, referred to as “goal-directed therapy” (252, 253). One conclusion from this approach is that those at very high risk of fracture should initiate treatment with an anabolic and subsequently transition to an antiresorptive, as the most effective sequence for fracture prevention (159, 180). A second is that clinicians should set BMD targets in each patient on the basis that achieved BMD on treatment is closely related to fracture risk during treatment (254, 255). However, this suggestion is confounded by the close relationship between BMD at the beginning and end of a treatment period, the correlation being 0.93 in 1 study (249). In an analysis of women treated with zoledronate over 6 years, it was the baseline BMD that was predictive of fracture (together with age and fracture history) not the change during treatment (249). In fact, current medications remain limited in their capacity to increase BMD so the possibility of returning most patients to near-normal values is low. Therefore, setting BMD targets currently is probably not of great value since our treatment options are limited to selecting which drug class we use first (anabolic vs antiresorptive) and, if we start with an anabolic, selecting which antiresorptive agent we transition to subsequently. The trial evidence indicates the principal antiresorptive agents have comparable antifracture efficacy, even though they vary in their antiresorptive potencies (see Fig. 1). Also, when using parenteral treatments, basing management decisions on changes in bone turnover markers or BMD is not evidence-based because our zoledronate studies found that these variables were not predictive of fracture in individuals either while receiving regular infusions or subsequently (77, 80). Therefore, goal-directed treatment remains an aspiration rather than a current reality.

## Treatment sequences and combinations

Osteoporosis is not a self-limiting condition unless there is a specific reversible cause, such as glucocorticoid use, which can be corrected. No AOM is curative, so long-term management is needed in patients at risk of fractures. Such management can involve sequences of various AOMs, periods off treatment (ie, drug holidays) when using long-acting agents, and the use of intermittent drug regimens such as zoledronate.

Alendronate has been the most widely used oral bisphosphonate over the past 30 years. The rare development of AFFs after long-term use has resulted in the introduction of drug holidays, commonly at 5 years, during which the risk of AFFs declines rapidly (124). As discussed previously, a 1- to 2-year drug holiday is recommended after alendronate, but a 6- to 12-month holiday may be better with risedronate because its antiresorptive effect is lost more rapidly. After 5 years' use, alendronate's effects can be maintained with half the standard dose (ie, 70 mg every 2 weeks) (57) but the effect of this dose reduction on AFF risk is unknown, so a drug holiday is preferable. If fracture risk justifies ongoing intervention at the end of the holiday, a further 5-year treatment course is usually instituted, though the incidence of AFFs in second courses has not been documented. An alternative strategy is to have alternating 2-year periods on and off alendronate since this maintains BMD satisfactorily (134, 256). If further treatment is not provided at the end of a holiday, fracture rates increase (103, 135). The FLEX study showed that continuing alendronate beyond 5 years reduced clinical vertebral fractures and, in women with femoral neck T-scores < −2.5, nonvertebral fractures also (257).

Zoledronate was administered annually in its 2 phase 3 trials, but more recent data indicate that zoledronate administration at 18-month intervals over 6 years achieves more effective prevention of nonvertebral fractures at lower cost and inconvenience to patients. It is likely that these benefits can be maintained with occasional dosing (eg, every 3 years).

In a patient initially treated with a bisphosphonate who has a suboptimal response (eg, further fracture, no significant increase in BMD), an anabolic agent is often considered. It is important to remember that AOMs only reduce fracture risk by 20% to 70% (Fig. 1), so a fracture on treatment does not necessarily represent treatment failure. Fractures on treatment are associated with high baseline fracture risk, frailty, weight loss, and falls (258), so these factors as well as bone-active medications should be reviewed. If a change in medication after an incident fracture is appropriate, teriparatide, abaloparatide, or romosozumab are all drugs that could be transitioned to, though this has been most studied with teriparatide. Cosman randomized osteoporotic women already taking either alendronate or raloxifene, to switch to teriparatide or to take teriparatide on top of their antiresorptive treatment for 18 months (259). Greater bone turnover increases were achieved by *switching* to teriparatide, whereas more positive BMD changes resulted from *adding* teriparatide. Fracture effects are unknown, but the BMD changes suggest that adding the anabolic is more attractive.

Langdahl randomized women already taking bisphosphonates for at least 3 years to transition to either teriparatide or romosozumab (154). In the teriparatide group, there were significant decreases in proximal femoral BMD at 6 months and progressive declines in femoral cortical BMD throughout the 1-year study. In those transitioning to romosozumab, proximal femoral BMD increased by 2.9% over 1 year. Lumbar spine BMD increased in both groups, 9.8% with romosozumab at 12 months and 5.4% with teriparatide. Fracture numbers were small and not significantly different between groups.

Following a course of an anabolic drug, patients are then transitioned to an antiresorptive treatment to “lock-in” the BMD gains from the anabolic. The FDA has now approved use of teriparatide for >24 months in those who remain at high fracture risk, so providing a second treatment with teriparatide is sometimes possible. Cosman provided a second course of teriparatide after an intervening year of alendronate, which increased spine BMD to a similar extent as seen during the original teriparatide course, so the total spine BMD increment was greater after 2 courses (260). Similarly, a second course of romosozumab produced some further increase in BMD, particularly at the spine (174, 261). The limited available data indicate that transition from teriparatide to romosozumab leads to BMD gain but the reverse transition does not (262).

Because of its rapid offset of effect, transitions from denosumab require care. Drug holidays are not appropriate after denosumab, and can cause clusters of vertebral fractures, as discussed previously. Transitioning from denosumab to a bisphosphonate has already been discussed. Switching from denosumab to teriparatide leads to a rapid loss of proximal femoral BMD (113), so is unhelpful. However, teriparatide plus denosumab has an additive effect on BMD (113), though its antifracture efficacy is unknown. A group of 16 patients who were treated for a year with denosumab then changed to romosozumab has been described, and they showed increasing spine BMD and stable hip BMD following that transition (263). Whether similar outcomes result after longer periods on denosumab before transitioning is not known. Because options for transitioning from denosumab are so limited, particularly in those in whom bisphosphonates are contraindicated, this issue should be carefully considered before this agent is prescribed.

**Figure 5:**
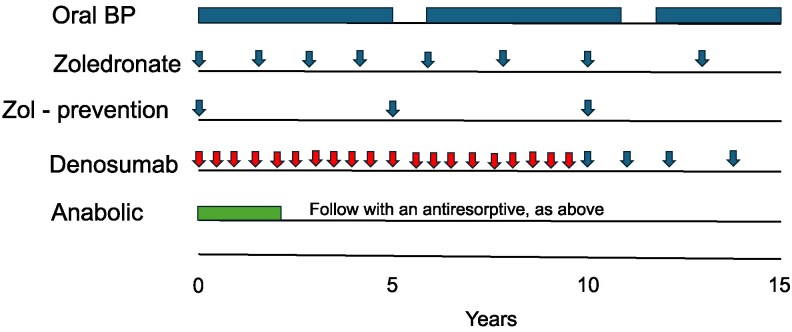
Commonly used sequences of interventions for management of osteoporosis. Periods of continuous drug administration are shown as solid bars, for oral bisphosphonates or anabolic drugs. The timing of periodic infusions of zoledronate or injections of denosumab are shown with vertical arrows.

The phase 3 studies of romosozumab used this drug for only 1 year before transitioning to an antiresorptive, either alendronate or denosumab. These sequences achieved antifracture efficacy that was superior to control. A small study also demonstrated that BMD gains from romosozumab are maintained for at least 2 years by a single infusion of zoledronate (175), providing a further attractive option. Some common sequences of AOM use are illustrated in Fig. 5.

It should be noted that evidence to support guidance for management beyond 5 to 10 years is very limited because gathering such evidence requires large, long-term, prospective, or retrospective studies that by their nature are difficult to conduct and expensive. Therefore, making recommendations for very long-term management requires extrapolation of the findings from current 5- to 10-year studies.

## Fracture liaison services

Ever since effective treatments for fracture prevention became available, there has been a failure to achieve uptake and adherence rates adequate to optimize their impact on the global burden of fractures. Typically, only about 20% of patients suffering a fragility fracture are subsequently started on AOMs (264). Fracture Liaison services have been developed as the major initiative to address this ongoing problem. They aim to identify patients presenting with fractures, identify risk factors for fracture, assess future fracture risk, and provide interventions (eg, AOMs, falls management) to mitigate this risk. They have been found to reduce fracture risk by about 30% beyond 2 years (265) and falls, and to be cost-effective (266). They are being increasingly adopted internationally, though not yet universally (267).

## Knowledge gaps and future research

Laboratory and clinical work over the past 40 years have produced a range of potent regulators of both bone formation and resorption. We have clear evidence of antifracture efficacy in those agents currently in use, but very limited data on their comparative efficacy, and even less on the wide range of treatment combinations and sequences that are possible, or the best options for treatment after the initial 5 to 10 years. Current indications are that novel therapeutic agents are unlikely to enter clinical practice in the foreseeable future, so the immediate research need is to optimize the use of the effective agents that we currently have available.

In recent years, some studies have compared the BMD effects of various sequences/combinations/durations, but differences in BMD effects do not necessarily predict differences in antifracture efficacy. For example, long-term use of denosumab clearly produces greater increases in BMD than does zoledronate but antifracture efficacy of these drugs is comparable. Similarly, the teriparatide phase 3 trial showed that the 40-µg dose increased spine and hip BMD substantially more than the 20-µg dose, but their effects on fracture were the same (153). Several authors have cited the meta-regression analyses of osteoporosis trials from Black et al (201) as evidence that antifracture efficacy and change in BMD are linearly related, though establishing that was not the intention of those analyses. If this relationship is analyzed only across the studies of effective agents, then BMD change and magnitude of antifracture efficacy are not related (201). This suggests that this might be a threshold phenomenon rather than a linear one, and that there is not a close relationship between marker and BMD changes on the 1 hand, and fracture prevention on the other, once a minimum BMD difference between groups has been achieved.

Therefore, we need appropriately powered, long-duration, studies of sequential (anabolic then antiresorptive or the reverse sequence) or combined (anabolic-antiresorptive vs single agents or sequences) treatment strategies with fracture endpoints. Osteoporosis therapeutics have substantially depended on industry funding to-date, but the increasing role of generic medicines will mean that these studies will probably require public-good funding. They may need to be multicenter to recruit the numbers needed to definitively demonstrate comparative antifracture efficacies between regimens.

Even the single agent, zoledronate, has much work still to be done. Superficially, dose intervals from 1 to 10 years appear to be equally effective, but these studies were carried out in very different populations. Because this drug is very attractive in its cost, convenience, and safety, further fracture studies of these dose intervals in early postmenopausal, osteopenic, and osteoporotic cohorts would be valuable.

Longevity continues to increase, so the burden of fractures in our aging population will also increase. Despite the departure of industry players from the center of the osteoporosis stage, the need for bigger and longer trials to establish the comparative efficacies in fracture prevention of present and novel treatment regimens will continue to demand our attention.

## Abbreviations

AFF: atypical femoral fracture
AOM: antiosteoporosis medicine
BMD: bone mineral density
CKD: chronic kidney disease
DXA: dual-energy X-ray absorptiometry
eGFR: estimated glomerular filtration rate
FDA: Food and Drug Administration
MOF: major osteoporotic fracture
NICE: National Institute for Health and Care Excellence
NNT: number needed to treat
ONJ: osteonecrosis of the jaw
SERM: selective estrogen receptor agonist
WHO: World Health Organization
